# Language switching and executive control in bilinguals with Mild Cognitive Impairment

**DOI:** 10.1002/alz.090671

**Published:** 2025-01-03

**Authors:** Marco Calabria, Anna Suades, Montserrat Juncadella, Jordi Ortiz‐Gil, Lidia Ugas, Isabel Sala, Alberto Lleo

**Affiliations:** ^1^ Faculty of Health Sciences, Universitat Oberta de Catalunya, Barcelona Spain; ^2^ ENTIA, Fundació de Neurorehabilitació i Recerca Cognitiva, Barcelona Spain; ^3^ FIDMAG Germanes Hospitalàries Research Foundation, Barcelona Spain; ^4^ Psychology Unit, Hospital General de Granollers, Granollers Spain; ^5^ Hospital Benito Menni, CASM, Sant Boi de Llobregat Spain; ^6^ Sant Pau Memory Unit, Hospital de la Santa Creu i Sant Pau ‐ Biomedical Research Institute Sant Pau ‐ Universitat Autònoma de Barcelona, Barcelona Spain; ^7^ Sant Pau Memory Unit, Hospital de la Santa Creu i Sant Pau, Biomedical Research Institute Sant Pau, Universitat Autònoma de Barcelona, Barcelona Spain

## Abstract

**Background:**

Bilingual Language Control (BLC) is a dynamic processing system that enables speakers to mitigate cross‐language interference while conversing and transitioning between languages. Research indicates that neurodegenerative diseases may compromise the effectiveness of language‐switching abilities in bilingual individuals, particularly those impacting the frontostriatal pathways. However, it remains unclear whether neurodegeneration affecting other neural pathways, such as Mild Cognitive Impairment (MCI), might influence the efficiency of BLC.

**Method:**

The study involved 40 patients diagnosed with MCI and 30 older adults. All participants were proficient bilinguals in Catalan and Spanish and early proficiency in both languages. Each participant engaged in a set of five tasks, including one task focused on language control (language switching) and four non‐linguistic control tasks: task switching, n‐back, Spatial Stroop, and flanker task.

**Result:**

MCI patients exhibited higher switch costs (switch minus repeat trials) compared to healthy controls, while demonstrating similar mixing costs (repeat minus single trials). Within the non‐linguistic control domain, MCI patients showed significant performance deficits only in the n‐back task when compared to controls. The regression analysis model, which incorporated the performance of non‐linguistic tasks as predictors for language switching performance, did not yield statistical significance.

**Conclusion:**

Notably, language control abilities may be compromised in bilingual patients even in the absence of neurodegeneration in brain regions traditionally linked to language selection and inhibition, such as the basal ganglia. Crucially, cognitive decline appears to have a more pronounced impact on language‐switching abilities than on the monitoring of the two languages. However, the existence of language control deficits does not correlate with executive control deficits, suggesting that these two control domains are not entirely overlapping.

